# Inducible expression of heat shock protein 20 protects airway epithelial cells against oxidative injury involving the Nrf2-NQO-1 pathway

**DOI:** 10.1186/s13578-020-00483-3

**Published:** 2020-10-19

**Authors:** Aihua Bao, Aying Ma, Hui Zhang, Lihua Qiao, Suqin Ben, Xin Zhou, Min Zhang

**Affiliations:** 1 Department of Respiratory and Critical Care Medicine, Shanghai General Hospital, Shanghai Jiao Tong University School of Medicine, 100 Haining Road, Shanghai, 200080 China; 2 Department of Respiratory and Critical Care Medicine, The First Affiliated Hospital of Zhengzhou University, Henan, China; 3 Department of Gynecology, The Fourth People’s Hospital of Shanghai, Tong Ji University, Shanghai, China

**Keywords:** Heat shock protein 20, Oxidative stress, Airway epithelial cell, Nrf2, NQO-1

## Abstract

**Background:**

Heat shock protein (HSP) 20 is a molecular chaperone that exerts multiple protective functions in various kinds of tissues. However, the expression of HSP20 and its specific functions in airway epithelial cells (AECs) remain elusive.

**Results:**

In current study, we first confirmed the inducible expression of HSP20 in mouse AECs and in a human bronchial epithelial cell line BEAS-2B cells, under different oxidant stressors. Then by establishing a HSP20-abundant mouse model with repeated low-level-ozone exposures and stimulating this model with a single high-level ozone exposure, we found that the HSP20 abundance along with its enhanced phosphorylation potentially contributed to the alleviation of oxidative injuries, evidenced by the decreases in the bodyweight reduction, the BAL neutrophil accumulation, the AECs shedding, and the BAL concentrations of albumin and E-cadherin. The biological function of HSP20 and its molecular mechanisms were further investigated in BEAS-2B cells that were transfected with *HSP20*-, unphosphorylatable *HSP20(Ala)* or empty vector plasmids prior to the stimulation of H_2_O_2_, of which its oxidant capacity has been proved to be similar with those of ozone in an air–liquid culture system. We found that the H_2_O_2_-induced intracellular ROS level and the early cell apoptosis were attenuated in the *HSP20-* but not *HSP20(Ala)-* transfected cells. The intracellular expression of NQO-1 (mRNA and protein) and the intranuclear content of Nrf2 were significantly increased in the *HSP20-* transfected cells but not in the *HSP20(Ala)*- and empty vector-transfected cells after the stimulation of H_2_O_2_.

**Conclusions:**

The inducible expression of HSP20 in AECs by oxidative stress exerts protective roles against oxidative damages, which may involve the activation of the Nrf2-NQO-1 pathway.

## Introduction

Oxidative stress plays an important role in the pathogenesis of chronic inflammatory airway disease, including chronic asthma and chronic obstructive pulmonary disease (COPD) [1]. Injuries caused by oxidants contribute to various aspects of the pathophysiological features of these chronic airway diseases. As the first line of host defense, airway epithelial cells (AECs) as well as their intercellular adjacent junctions are vulnerable to oxidative injuries, as they are frequently exposed to enormous kinds of airborne oxidants, such as ozone [2, 3]. The survival of AECs under oxidant insults relies on the intracellular redox homeostasis, which is maintained by the balance between reactive oxygen species (ROS) and antioxidant enzymes [4]^.^ Enhancing the antioxidant capacity of AECs bears promising potentials to be a candidate therapeutic target [5].

Antioxidant enzymes like NAD(P)H quinone oxidoreductase 1 (NQO-1), and glutathione S-transferases (GST) are sensitive to exogeneous oxidants [6, 7]. The gene expressions of these antioxidant enzymes are regulated by a transcription factor, NF-E2 related factor 2 (Nrf2), which will translocate into the nucleus upon the stimulation of oxidant signals and bind the antioxidant response element (ARE) in the promoter region of genes of these enzymes [6]. Thus, the activity of this Nrf2-NQO-1 pathway is considered to be a vital component of antioxidant capacity in mammalian cells.

Small heat shock proteins (sHSPs) belong to a large group of proteins that was initially discovered in different Drosophila tissues during recovery following a transiently mild and sublethal increase of normal core body temperature [7]. Other unfavorable conditions like hypoxia, ischemia, and oxidative stress can also induce the synthesis of these small HSPs, which further exert protective roles in multiple tissues against these insults [8]. The biological protective functions of small HSPs have gained an increasing body of attention.

HSP20, also named as HSPB6, is a broadly expressed small HSP found in various kinds of tissues [9] and exerts multifunctional protective roles in multiple organs [10]. For instance, HSP20 was proved to be protective against a number of brain diseases including cerebral amyloid angiopathy [11], Alzheimer disease [12] and forebrain ischaemia [13]. The protective role HSP20 was also illustrated in cardiovascular diseases, such as ischaemia–reperfusion [14, 15] and doxorubicin cardiotoxicity [16]. Due to these protective effects in the milieu of oxidative stress, HSP20 is considered an anti-oxidant agent [10, 17]. Previous studies have demonstrated that HSP20 is biophysically screened as a small molecular target for the treatment of chronic obstructive pulmonary disease (COPD), a very common chronic oxidative stress-related airway disease [18]_._ However, it is by far not clear whether it is expressed and functions in the AECs.

We thus investigated the expression as well as the role of HSP20 in AECs both in vivo and in vitro*.* The results of current study indicate that HSP20 can be induced to express and exerts protective roles against oxidative injuries in AECs, implicating HSP20 a potential therapeutic target for oxidative stress-related airway diseases.

## Results

### The stable abundant expression of HSP20 in AECs of mice preconditioned with repeated low-level-ozone exposures

**Fig. 1:**
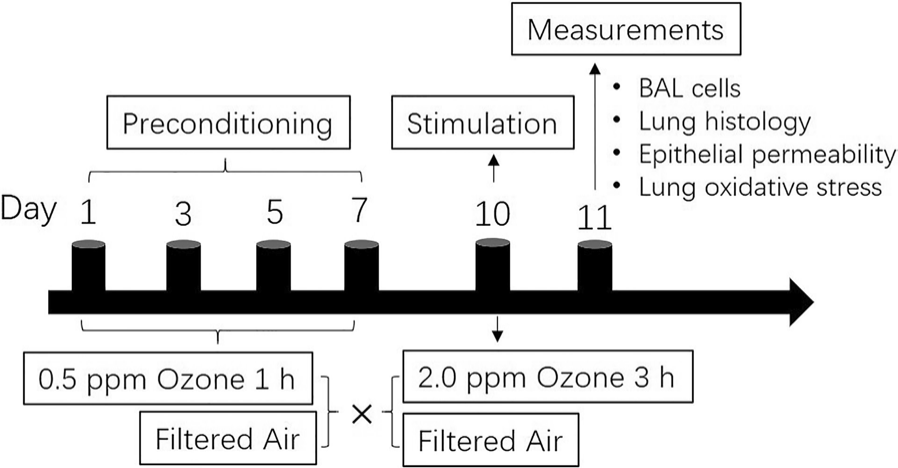
Schematic diagram of animal experimental protocol. Mice were preconditioned with 0.5 ppm O_3_ or filtered air for 1 h at day 1, 3, 5 and 7. On day 10 (48 h after the last preconditioning), mice were further stimulated with 2.0 ppm O3 or filtered air for 3 h. On day 11, biological measurements including differential BAL cell counting, lung histological evaluation, epithelial permeability and lung oxidative stress were performed

**Fig. 2:**
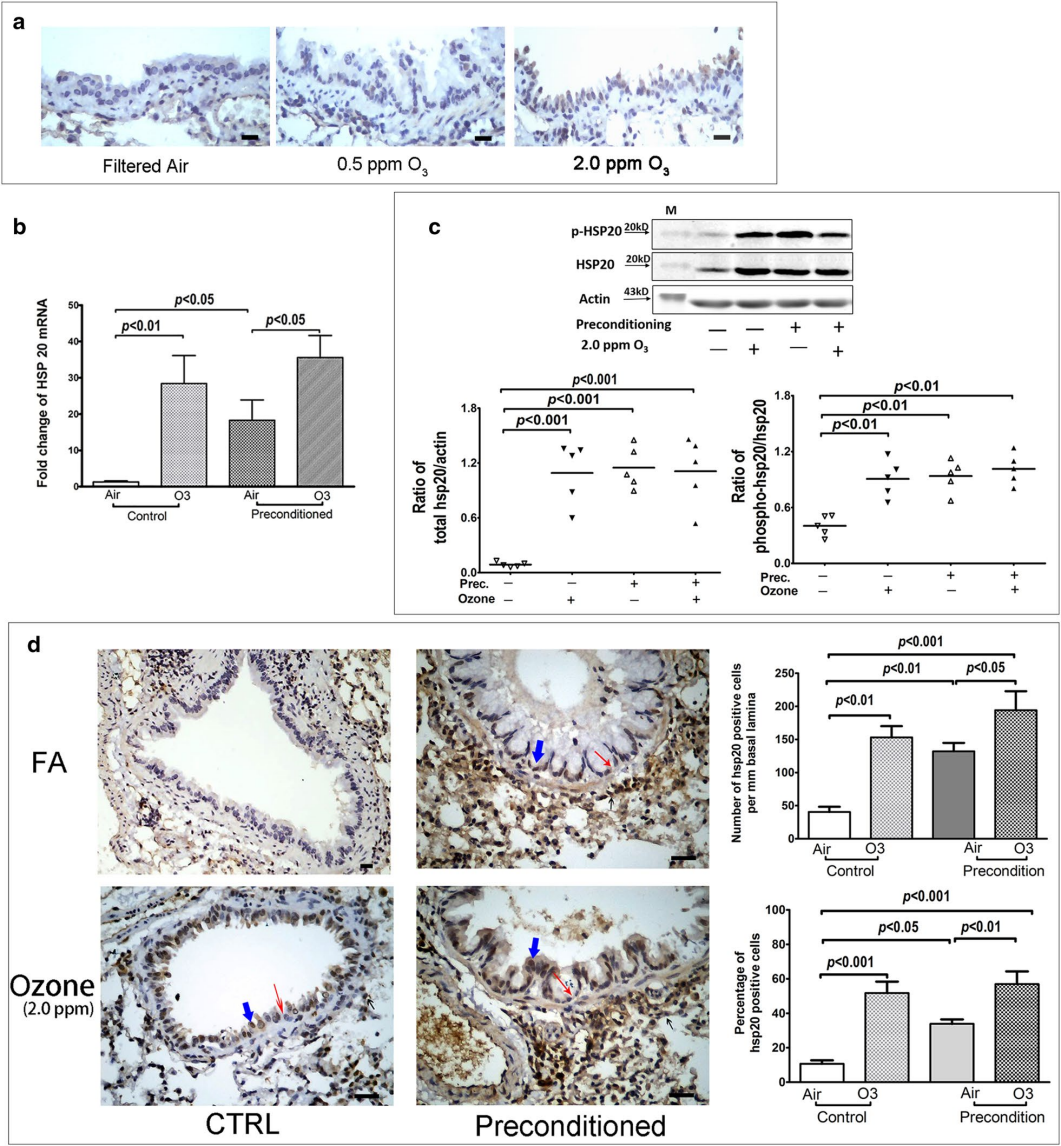
The expression of HSP20 in AECs of mice in vivo. The inducible expression of HSP20 by oxidative stress in mouse AECs in vivo was tentatively explored in a preliminary experiment, where mice (n = 3) were exposed to filtered air, 0.5 and 2.0 ppm ozone, for 1 h (h) and their lungs were harvested 24 h after the exposure. **a** The IHC staining of HSP20 on lung tissue sections from preliminary experiment (black bar represents 50 μm). Then the expressions of HSP20 in AECs and lungs of an animal model that preconditioned with repeated low-level ozone exposures (0.5 ppm) were evaluated at protein and mRNA level. **b** HSP20 mRNA expression in lung tissues measured with RT-qPCR. **c** The expression of total and phosphorylated HSP20 protein in lung tissues measured with Western blot. The ratio of total HSP20 protein with actin (bottom left panel) and the phosphorylated- with the total- HSP20 protein (bottom right panel) were calculated based on their densitometry data, respectively. **d** The local expression of HSP20 on lung tissues were detected by IHC, and the typical pictures were presented (left panel) (the black bar represents 50 μm). The number of HSP20 positive cells per micrometer basal lamina (upper right panel) and the percentage of HSP20 positive cells (lower right panel) were counted and calculated on the segments of distant bronchi in HSP20-IHC stained lung sections. Data are presented as mean ± SD for n = 5 in each group. *Prec.* preconditioning, *FA* filtered air. Green wide arrow indicating HSP20-positive cells, red thin arrow indicating epithelial cell shedding, black short arrow indicating inflammatory cells

In the pilot experiment, IHC staining showed that HSP20 expression was induced in mouse AECs after different level of ozone exposures, even at a level as low as 0.5 ppm (Fig. 2a). Based on this observation, we postulated that the expression of HSP20 in mouse AECs could be stably induced while collateral injuries remaining minimum if mice were repeatedly exposed to a very low level of ozone. To verify this postulation, we measured the mRNA and protein expressions of HSP20 in lungs of low-level-ozone (0.5 ppm) preconditioned mouse model, and compared with those preconditioned with filtered air (referred to air-exposed control data vs. air-exposed preconditioned data in Fig. 2). RT-qPCR showed that HSP20 mRNA expression in lungs was significantly promoted in these preconditioned mouse models, in comparison with the control mice (Fig. 2b). Western blot analysis revealed a universal significant increase in both the total and the phosphorylated HSP20 proteins in lungs of the preconditioned mouse models, but not of the control mice (Fig. 2c). This was also the case for the local expression of HSP20 in mouse AECs, demonstrated by IHC staining (Fig. 2d). Of note, in these mouse models, the induced HSP20 expressions were evaluated 96 h (d7–d11, Fig. 1) after the last low-level-ozone exposure, evidencing the stable abundance of HSP20 therein, which qualified these preconditioned mice as a HSP20-abundant animal model to serve as an in vivo gain-of-function system and facilitate the study of the biological actions of HSP20.

We thus applied one single high-level-ozone (2.0 ppm) exposure (3 h) as an oxidant insult to this HSP20-abundant mouse model to investigate the biological functions of HSP20 in this scenario. To this end, we first evaluated the corresponding effects of this single exposure on the lung HSP20 expressions, in control or model mice, respectively (referred to O_3_-exposed control data vs. O_3_-exposed preconditioned data in Fig. 2). Data showed that this single high-level-ozone exposure significantly increased the HSP20 mRNA expression in both the HSP20-abundant mouse model and the control mice (Fig. 2b). While for the total and the phosphorylated protein of HSP20, they were only increased by this high-level-O_3_ stimulation in the control mice but not in those HSP20-abundant mouse models (Fig. 2c). As to the local HSP20 expressions in AECs, they were increased by the subsequent high-level-O_3_ stimulation similarly in the control mice and the HSP20-abundant mouse models (Fig. 2d).

### *The *in vivo* protective function of HSP20 on AECs of the HSP20-abundant mice from oxidative injuries*

The influences of HSP20 abundance on the high-level-ozone-induced biological effects were evaluated, from aspects including ozone-induced toxicity, inflammation and oxidative stress.

**Fig. 3:**
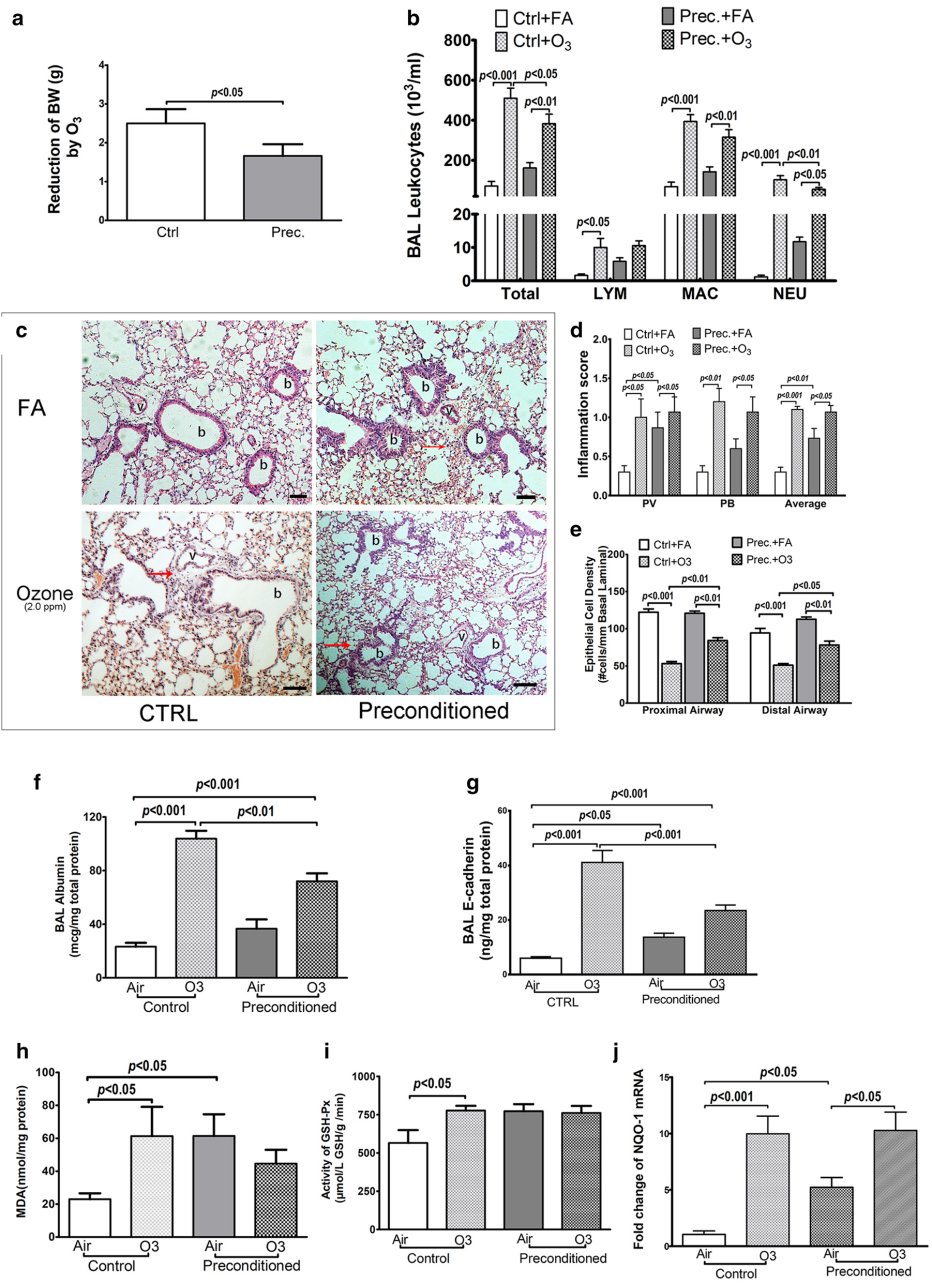
The in vivo protective function of HSP20-abundance against oxidative damages. Mice were repeatedly exposed to 0.5 ppm O_3_ (preconditioned, n = 10) or filtered air (FA) (control, n = 10) for 1 h on every other day for 7 days. Forty-eight hours after the last exposure, equal number (n = 5) of preconditioned or control mice were stimulated with 2.0 ppm O_3_ or FA for 3 h, and BAL fluid samples and lung tissues were harvested 24 h after the stimulation. **a** Mice bodyweight before and after the high-level-O3 stimulation. **b** Total and differential cell counts in BAL fluid samples. **c** Typical pictures of H&E staining on lung sections (Black bar represents 40 μm, red arrow indicating inflammatory cells, b = bronchi, v = vessel). **d** Histological inflammation in perivascular (PV), peribronchial (PB) area and their average. **e** Airway epithelial cell density on basal lamina of distant bronchi on H&E stained lung sections. **f**, **g** The concentration of Albumin and E-cadherin in cell-free BAL fluid. **h**, **i** The content of MDA and the activity of GSH-Px in lung tissue homogenization. **j** The mRNA expression of NQO-1 in lung tissues. Data are expressed as mean ± SD. BW: body weight; Prec.: preconditioning; FA: filtered air

Exposure to ozone can reduce the bodyweight of mice, which indicates the toxicity of ozone [19]. In current study, the reduction of bodyweight induced by high-level-ozone stimulation was less prominent in the HSP20-abundant mouse models than those in the control mice (Fig. 3a).

Differential cell counts on BALF samples showed similar cellularities between the control mice and the HSP20-abundant mouse models upon stimulation with filtered air (Fig. 3b). The single high-level-ozone stimulation increased the number of total leukocytes, macrophages and neutrophils in the BALF samples of both groups of mice, with less increments of total leukocytes and neutrophils observed in the HSP20-abundant models (Fig. 3b). Notably, this high-level-ozone stimulation increased the BAL lymphocytes only in the control mice but not in the HSP20-abundant mouse model (Fig. 3b).

Histological evaluation on H&E-stained lung sections showed that upon stimulation with filtered air, the HSP20-abundant mouse models had higher inflammation scores in the perivascular area as well as in average, than the control mice. Stimulation with high-level-ozone, however, increased the average inflammation scores and those in both the perivascular and the peribronchial area to a similar level between the control mice and the HSP20-abundant mouse models (Fig. 3d).

The ozonic effects on epithelial cells were evaluated on two parameters: one is the epithelial cell density, which is inversely associated with the AECs shedding [20]; another is the epithelial permeability, evaluated by the changes of albumin concentration in BALF, reflecting the integrity of epithelial barrier [21].

Figure 3f shows that the epithelial cell density of both the proximal and distal airways was similar between the control mice and the HSP20-abundant mouse models when stimulated with filtered air. High-level-O_3_ stimulation decreased AECs density in the HSP20-abundant mouse models to an extent less than those observed in the control mice (Fig. 3e)

The level of BAL concentration of albumin was similar between the control mice and the HSP20-abundant mouse models under the stimulation of filtered air. When stimulated with high-level-O_3_, the HSP20-abundant mouse models had lower albumin level in BALF samples than those of the control mice (Fig. 3f).

To confirm the change of epithelium permeability that reflected by albumin leakage, we also measured the BAL concentration of E-cadherin, a key component of adherent junction of airway epithelium that serves as a marker for epithelium integrity [22]. When exposed to filtered air, HSP20-abundant models had higher BAL concentration of E-cadherin than those of the control mice (Fig. 3g). While stimulation with high-level-O_3_ increased the BAL concentration of E-cadherin to a higher extent in the control mice than those of the preconditioned mice, leading to a lower BAL concentration of E-cadherin in the HSP20-abundant models (Fig. 3g).

Oxidative stress was evaluated in lung homogenates by measuring the content of malondialdehyde (MDA) and the activity of glutathione peroxidase (GSH-Px). Mice of HSP20-abundant model had higher level of MDA content and GSH-Px activity than those of control mice upon stimulation with filtered air. However, high-level-ozone stimulation significantly increased the level of both index in lungs of the control mice but not in the HSP20-abundant mouse models (Fig. 3h, i). To further understand the abovementioned phenomenon, we also measured the mRNA level of NQO-1, an antioxidant enzyme, in lungs of mice with RT-qPCR. We found that after stimulation with filtered air, mice in HSP20-abundant model had significantly higher level of NQO-1 mRNA than control mice. While stimulation with high-level-ozone increased the expression of NQO-1 mRNA to a similar level in lungs between the control mice and the HSP20-abundant mouse models (Fig. 3j).

### ***The comparable ***in vitro*** effects of ozone and H***_***2***_***O***_***2***_*** on oxidative stress and the inducible expression of HSP20 in human airway epithelial cells***

**Fig. 4:**
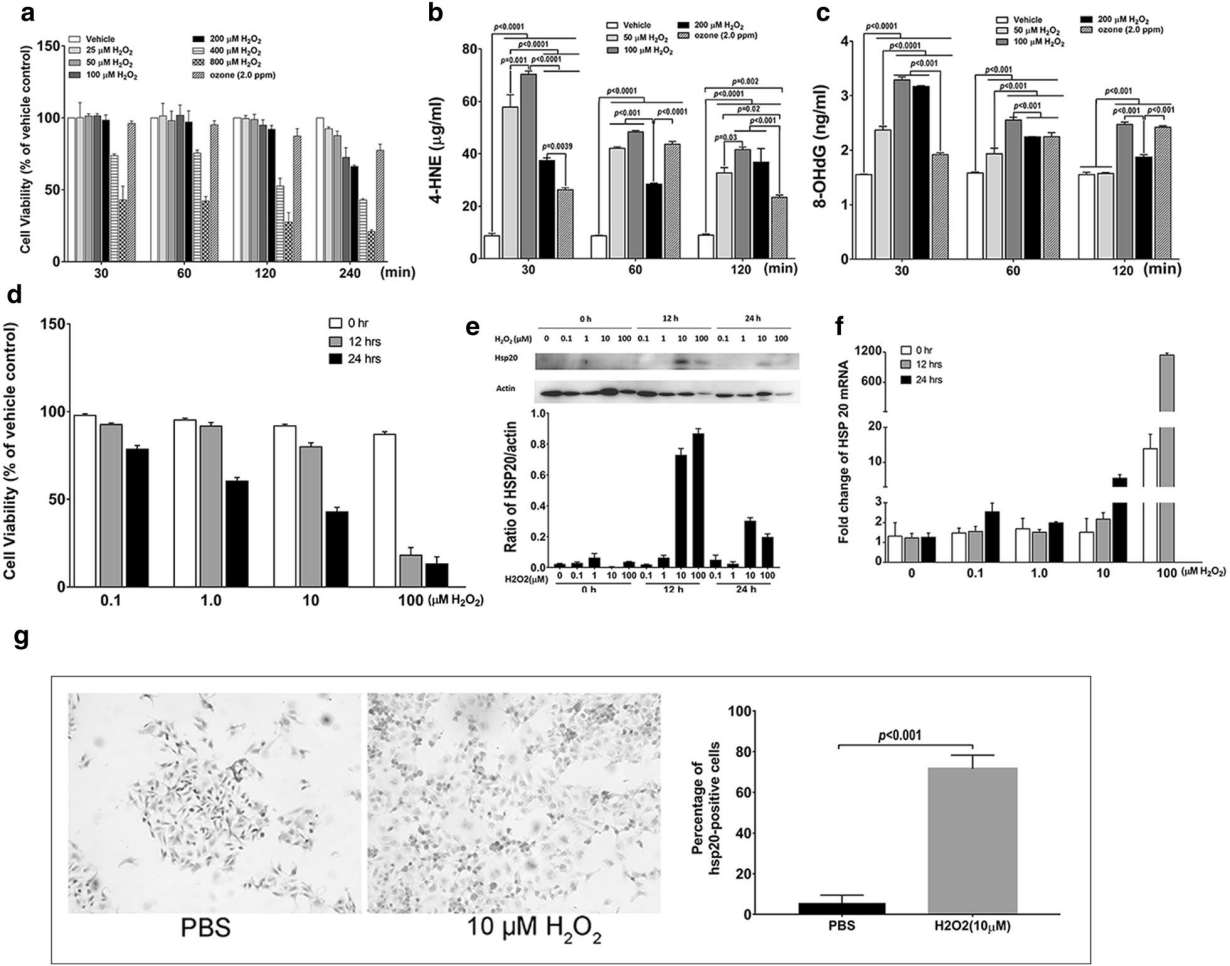
The comparable effects of ozone and H_2_O_2_ and the induced-expression of HSP20 in human AECs. Human bronchial epithelial cell line 16-HBE were cultured in air–liquid-interface. **a** Cell viability was measured with MTS after being stimulated with 2.0 ppm ozone or series of hydrogen peroxide (H_2_O_2_) (0, 25, 50, 100, 200, 400, 800 μM) for 0.5, 1, 2, 4 h. **b** 4-Hydroxynonenal (4-HNE) in supernatants and **c** 8-hydroxy-deoxyguanosine (8-OHdG) in DNA extracts were measured with ELISA in 16 HBE cells stimulated with 2.0 ppm ozone or series of hydrogen peroxide (H_2_O_2_) (0, 50, 100, 200 μM) for 0.5, 1, 2 h, respectively. BEAS-2B cells were cultured in submergence and stimulated with a series of H_2_O_2_ (0, 0.1, 1.0, 10, 100 μM) for 1 h and then were harvested 0, 12, and 24 h later. Cell viability was evaluated with MTT (D). **e**, **f** The mRNA and protein expression of HSP20 were measured with Western blot (same volume of protein sample added to gels) and RT-qPCR (same amount of DNA sample added to microplates), respectively. These measurements were performed with triplicates. **g** The immunocytochemistry (IHC) staining for HSP20 on BEAS-2B cells stimulated with 10 μM H_2_O_2_ (PBS as control) for 1 h and harvested 12 h after. Data are presented as mean ± SD

The in vivo experiments suggest the possible contribution of HSP20 to the protective capacity of the HSP20-abundant mouse model against O_3_-related injuries in mouse airway epithelium. We thus performed a series of in vitro experiments to further substantiate this possibility on human cell lines. As ozone is frequently used for in vivo oxidative stimuli and H_2_O_2_ for in vitro stimuli, we first explored whether the in vitro effects of these two stimuli were comparable, via measuring cell viability and oxidants metabolisms in ALI-cultured 16HBE cells. MTS assay showed that when stimulated for 30, 60 and 120 min, cell death caused by 2.0 ppm ozone were similar with those caused by 50, 100 and 200 μM H_2_O_2_. When exposing for 240 min, 2.0 ppm ozone caused approximately 30% cell death, close to 100 μM H_2_O_2_ (Fig. 4a). For the effects on the production of oxidant metabolites, ozone elicited lower production of 4-HNE and 8-OHdG than those induced by different H_2_O_2_ exposures in cells exposed for 30 min. When the exposure duration came to 60 min, ozone-induced production of 4-HNE was similar with those induced by 50 and 100 μM H_2_O_2_ but higher than those induced by 200 μM H_2_O_2_, while the ozone-induced production of 8-OHdG was higher than those caused by 50 μM, lower than those by100 μM and similar with those by 200 μM H_2_O_2_, respectively. When cells were exposed for 120 min, ozone-induced production of 4-HNE was lower than those induced by different H_2_O_2_ exposures, while the ozone-induced production of 8-OHdG was higher than those induced by 50 μM and 200 μM but similar with those cased by 100 μM H_2_O_2_ (Fig. 4b, c).

After we confirmed the comparable in vitro effects of ozone and H_2_O_2_, we conducted a preliminary study to verify the inducible expression of HSP20 in human bronchial epithelial cells.

In the preliminary experiment, MTT assay showed that H_2_O_2_-caused cell death were more prominent under the stimulation of 100 μM H_2_O_2_ for 12 and 24 h (83 and 87%, respectively) (Fig. 4 D). Western blot assay showed that the HSP20 protein was most abundant in cells stimulated with 10 μM H_2_O_2_ and detected at 12 h (Fig. 4e). RT-qPCR measurements also revealed a twofold increase of HSP20 mRNA in cells stimulated with 1.0 μM H_2_O_2_ at 24 h and 10 μM H_2_O_2_ at 12 h (Fig. 4f). Accordingly, we chose 10 μM as the optimal H_2_O_2_ concentration for stimulation and 12 h as the optimal detecting timing for HSP20 expression in subsequent experiments, including the one confirming the expression of HSP20 protein in BEAS-2B cells using immunocytochemistry (ICC) staining (Fig. 4g).

### *The *in vitro* protective effect of the pEX-3-HSP20 transfection on BEAS-2B cells against the oxidative stress*

To reveal the biological function of HSP20 in vitro, BEAS-2B cells were transfected with *HSP20*-overexpressed plasmid (pEX-3-*Hsp20*)*,* unphosphorylatable *HSP20* mutant plasmid *(*pEX-3-*Hsp20(Ala)),* and vector plasmid (pEX-3), respectively. All cells were stimulated with 10 μM H_2_O_2_ and harvested in 12 h.

**Fig. 5:**
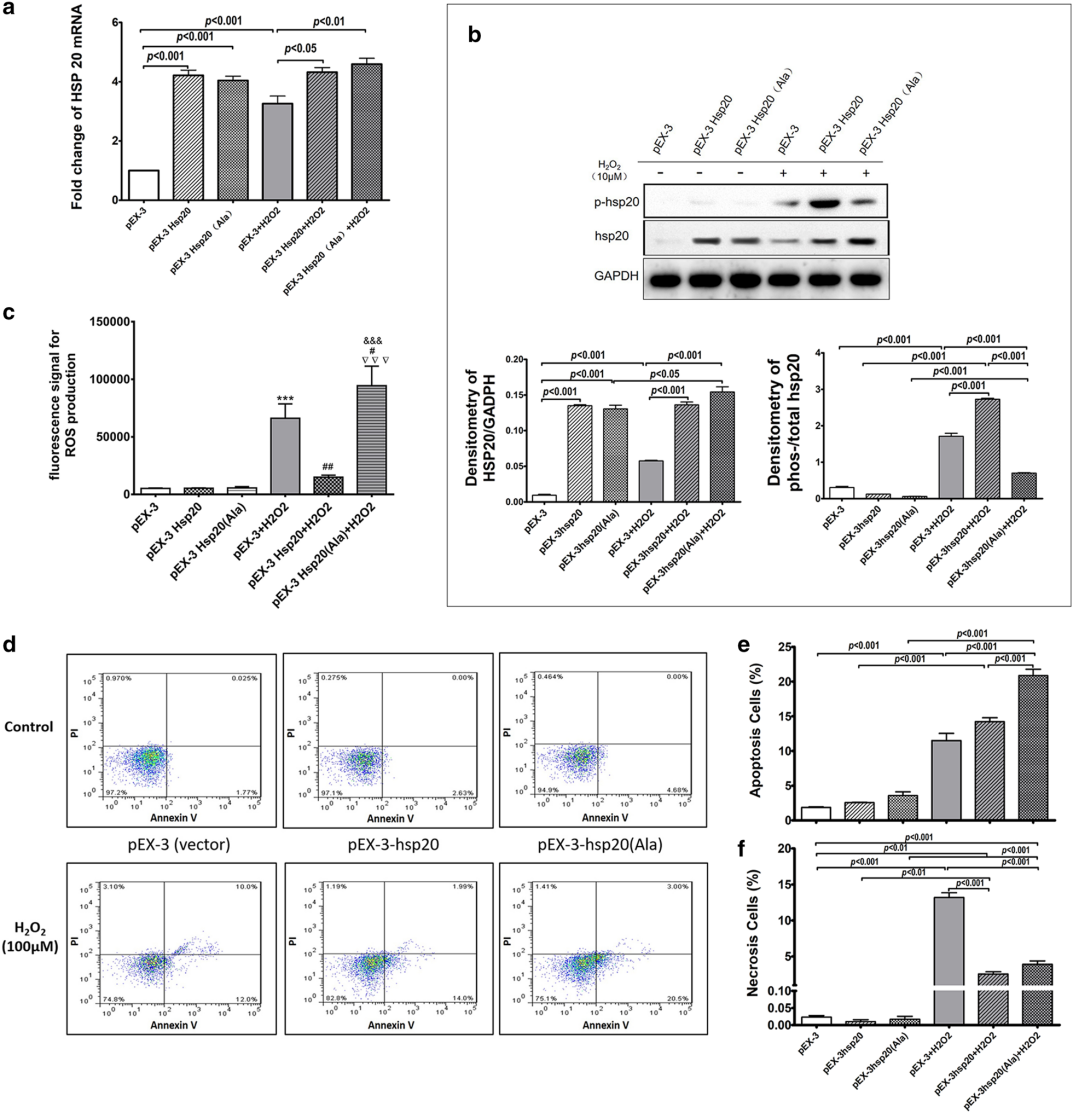
The in vitro protective effect of HSP20 on BEAS-2B cells. BEAS-2B cells were transfected with pEX-3, pEX-3-*HSP20*, and pEX-3-*HSP20(Ala)* and then stimulated with H_2_O_2_ (10 μM) for 1 h. **a** The expression of HSP20 mRNA measured with RT-qPCR. **b** The phosphorylated and total HSP20 protein measured with Western blot. **c** The intracellular content of reactive oxidative species (ROS) measured with DCFH-DA-based method. **d** Cell apoptosis measured with FACS counting on the Annexin V-FITC and PI stained cells and the early cell apoptosis (**e)** and cell necrosis (including late cell apoptosis) (**f**) were calculated. Data are expressed as mean ± SD

RT-qPCR measurements showed that the expressions of HSP20 mRNA were elevated to similar extent in the pEX-3-*HSP20-* as well as the pEX-3-*HSP20(Ala)-* transfected cells but not in the pEX-3- transfected cells at the baseline level. Stimulation with H_2_O_2_ significantly promoted the HSP20 mRNA expression in pEX-3- but not in pEX-3-*HSP20-* and pEX-3-*HSP20(Ala)-* transfected cells (Fig. 5a). Western blot assay revealed that the content of total HSP20 protein of the pEX-3-*HSP20-* as well as the pEX-3-*HSP20(Ala)-* transfected cells were both higher than those in vector (pEX-3)-transfected cells at the baseline level. Stimulation with H_2_O_2_ increased the total HSP20 protein expression in the pEX-3- and the pEX-3-*HSP20(Ala)-* transfected cells but not in the pEX-3-*HSP20-*transfected cells (Fig. 5b). The ratio of phosphorylated-/ total- HSP20 protein, were similarly low in all three groups of cells at the baseline level. Subsequent H_2_O_2_ stimulation significantly increased the ratio of phosphorylated-/ total- HSP20 in all three groups of cells, while in pEX-3*-HSP20-* transfected cells this ratio was significantly higher than those in the pEX-3- and the pEX-3*-HSP20(Ala)-* transfected cells, respectively (Fig. 5b).

The influences of HSP20 on intracellular oxidative stress metabolism and cell survival were evaluated as well. Data showed that the level of intracellular ROS was universally low in all three groups of cells at baseline level. Stimulation with H_2_O_2_ significantly increased the intracellular ROS level in the pEX-3- and the pEX-3-*HSP20(Ala)-* transfected cells but not in pEX-3-*HSP20-*transfected cells (Fig. 5c).

The cell apoptosis assay showed that the extent of early cell apoptosis induced by the H_2_O_2_ was similar in vector (pEX-3)- and pEX-3-*HSP20-* transfected cells, but significantly higher in pEX-3-*HSP20(Ala)*-transfected cells. However, the extent of the cell necrosis (including late cell apoptosis) induced by the same H_2_O_2_ stimulation was prominent in vector (pEX-3)- transfected cells, but not in the pEX-3-*HSP20-* and the pEX-3-*HSP20(Ala)-* transfected cells (Fig. 5d, f).

### The influence of HSP20 and its phosphorylation on the Nrf2-NQO-1 expression

**Fig. 6:**
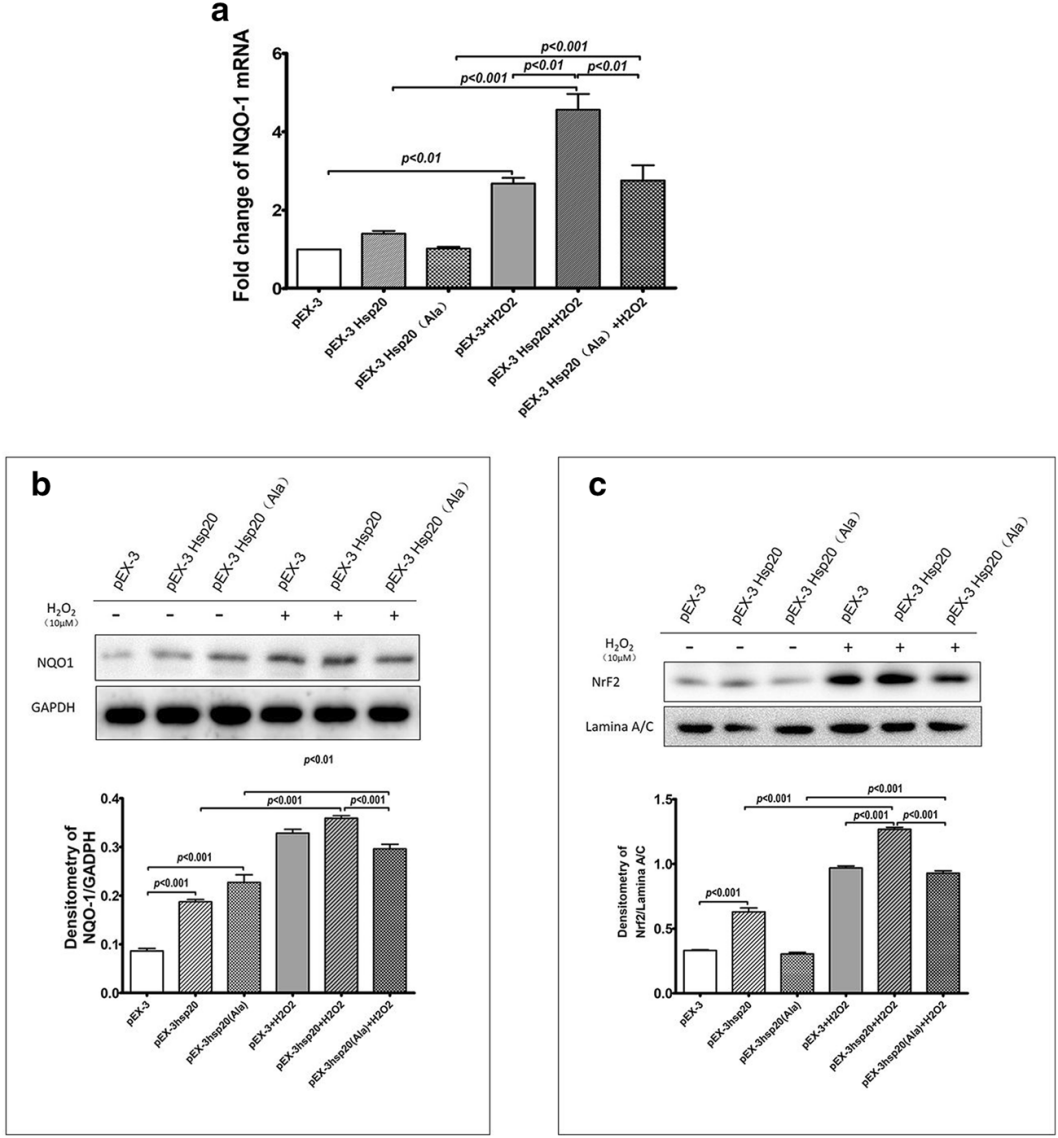
The influence of HSP20 and its phosphorylation on the Nrf2-NQO-1 expression in BEAS-2B cells. BEAS-2B cells were transfected with pEX-3, pEX-3-*HSP20*, and pEX-3-*HSP20(Ala)* and then stimulated with H_2_O_2_ (10 μM) for 1 h. **a** The mRNA expressions of NQO-1 measured with RT-qPCR. **b** The protein expression of NQO-1 and GADPH (control) in whole cell lysis and **c** Nrf2 and Lamina A/C (control) in nuclear extractions were measured with Western blot, respectively. Data are expressed as mean ± SD on three independent experiments

To further understand the potential influence of HSP20 on the metabolism of oxidative products, we measured the expression of NQO-1 and Nrf2 in differently transfected cells with and without the H_2_O_2_ stimulation, respectively. The NQO-1 mRNA expression level was similar in all three groups of unstimulated transfected cells, while the stimulation of H_2_O_2_ increased the NQO-1 mRNA expression threefold in the pEX-3- and the pEX-3-*HSP20(Ala)*- transfected cells and fivefold in the pEX-3-*HSP20-*transfected cells (Fig. 6a). Western blot measurements showed that without the stimulation of H_2_O_2_, the intracellular content of NQO-1 protein in both the pEX-3-*HSP20-* and the pEX-3-*HSP20(Ala)-* transfected cells were higher than those in the pEX-3-transfected cells. The stimulation of H_2_O_2_ significantly increased the intracellular content of NQO-1 protein in all three groups of cells, while the level of NQO-1 protein expression in the pEX-3-*HSP20*-transfected cells was significantly higher than those in the pEX-3-*HSP20(Ala)*-transfected cells (Fig. 6b).

To illustrate the underlying mechanisms of the promoting effect of HSP20 on the expression of NQO-1, we further investigated the nuclear accumulation of Nrf2, which is an upstream regulator of NQO-1. Western blot data showed that without the H_2_O_2_ stimulation, the intranuclear content of Nrf2 protein was elevated only in the pEX-3-*HSP20-*transfected but not in the pEX-3-*HSP20(Ala)-* and the pEX-3- transfected cells. The stimulation of H_2_O_2_ significantly increased the intranuclear Nrf2 in all three groups of cells, but more prominent in the pEX-3-*HSP20*-transfected cells, than those observed in the pEX-3- and the pEX-3-*HSP20(Ala)*- transfected cells (Fig. 6c).

## Discussion

As a broadly expressed small heat shock protein, HSP20′s expression and its biological functions in AECs are currently unknown. In current study, by using in vivo and in vitro models, we demonstrated that HSP20 can be induced by oxidative stresses in mouse and human AECs, which can in turn protect AECs from oxidative injuries through mechanisms that involve the Nrf2-NQO-1 pathway.

To our knowledge, there is no direct evidence showing the expression of HSP20 in AECs. In the in vivo experiment of current study, we found that at baseline level, the expression of HSP20 in whole lung tissues was abundant, as showed in the Western blot assay (Fig. 2c), but almost undetectable locally in AECs, as showed in the IHC test (Fig. 2d). While after stimulation with oxidant ozone, the expression of HSP20 in AECs was drastically increased. This observation was consistent with a fact that HSP20 is constitutively expressed in airway smooth muscles [23] and demonstrated the inducible expression of HSP20 in AECs. Though HSP20 belongs to small heat shock protein family, it is actually incapable of being induced by heat [9, 24]. Instead, some stress conditions, like congestive heart failure and exercise training, can increase the HSP20 level in the dog’s and rat’s hearts [25, 26]. In the current study, we demonstrated that the expression of HSP20 in mouse or human AECs can be induced by oxidative stress, which is consistent with other studies reporting the inducible expression of HSP20 by H_2_O_2_ stimulation [27] or ultraviolet B radiation [28] in the monogonont rotifer.

Driven by the unknown biological functions of HSP20 in AECs, a series of in vivo and in vitro experiments were conducted in current study. With measurements on the comprehensive parameters including systemic changes, like the bodyweight loss; and local changes, like inflammation in airways and lung compartments and damages in airway epithelium, data of the in vivo experiments showed that the detrimental effects elicited by high-level-ozone were significantly and comprehensively mitigated in the HSP20-abundant mouse model. Mitigation on these ozonic effects indeed reflects a protective property of the HSP20 abundance in these preconditioned animal models. Furthermore, these data for the first time showed that HSP20 may bear potentials to be protective not only in AECs against cellular damages, but also beneficial for the epithelial integrity, through maintaining the epithelial barrier and stabilizing epithelial junctions under potent oxidative stress.

In addition to the influences on the ozonic damages, the abundance of HSP20 in the preconditioned mouse model subsided the oxidants metabolisms, such as the MDA production and the GSH-Px activity, and increased the expression of an antioxidant enzyme NQO-1 in lung tissues as well. These data gave us a clue that HSP20 may possess certain degree of antioxidant properties. Of note, preconditioning of ozone has long been reported to be protective in animal organs against various kinds of oxidative insults in rats, such as liver ischaemia–reperfusion (I/R) injury [29], cisplatin-induced nephrotoxicity [30], renal transplantation [31], etc. Most of these studies reported the protective functions of ozone preconditioning were biophysiologically attributed to the maintenance of redox balance, of which the underlying mechanisms remain elusive. Current study also added to the literature a point of view that HSP20 might have contributions to maintaining the redox balance in ozone preconditioned animal models, which requires future studies.

Admittedly, in these in vivo experiments, though we have demonstrated the stable abundant expression of HSP20 in AECs of the low-level-ozone-preconditioned mouse model with different methodologies, the current animal model is, compared with the traditional gain-of-function animal model established with genetic manipulation, more complicated in regards of biological background and hence less convincing in demonstrating the biological effects of HSP20. Besides, the exact evaluation of local HSP20 expression in AECs was not satisfactorily fulfilled in current model, and more precise approaches such as Laser capture microdissection technology would achieve more in this regard. Thus, these tentative and pilot data require to be validated by in vitro investigations and future in vivo studies utilizing more sophisticated technologies and genetically modified animal models.

To further substantiate and explore the underlying mechanisms of the findings from the in vivo experiment, we performed a series of in vitro experiments, where HSP20 expression and its phosphorylatability were genetically modulated in BEAS-2B cells. As expected, overexpression of HSP20 inhibited the H_2_O_2_-induced cell necrosis (or late cell apoptosis), demonstrating the protective effects of HSP20 against the oxidative damages, which is consistent with current in vivo findings. Furthermore, the phosphorylation of HSP20 is key to its protective function, for the observation that overexpression of the HSP20 unphosphorylatable mutant fail to inhibit the cell apoptosis induced by H_2_O_2_. Consistently, several studies have previously demonstrated the essential role of phosphorylation for HSP20 to exert protective functions in multiple tissues [17, 23].

It has been reported that phosphorylated HSP20 exerts anti-apoptosis effects through mechanisms including protein phosphatase 1 [32], NF-kappa B [33], and Bcl-2/Bax [14]. Our data added to the literature by showing the potential contribution of intracellular ROS to the anti-apoptotic effect of phosphorylated HSP20, for the intracellular ROS level was decreased in cells undergone HSP20 but not its unphosphorylatable mutant overexpression. As a well-documented fact, ROS activation of JNK can induce both extrinsic and intrinsic apoptotic signaling [34, 35], of which this process is associated with decreased cellular GSH levels and the loss of cellular redox balance [35, 36]. Therefore, inhibition on the intracellular ROS by phosphorylated HSP20 may contribute to its anti-apoptosis effects.

How HSP20 regulates the intracellular metabolism of ROS in AECs is an intriguing question. It has been well-documented that Nrf2-NQO-1 pathway play crucial roles in manipulating the intracellular metabolisms of ROS. In our experiment, we did observe consistent increases in the intracellular NQO-1 protein and mRNA, as well as the intranuclear Nrf2 protein in cells undergone HSP20 overexpression after the H_2_O_2_ stimulation, with the extent of these increases being significantly higher than those in cells received either HSP20 unphosphorylatable mutant or blank vector. It is very possible that HSP20 might promote the translocation of Nrf2 from cytoplasm to nuclear, which further activates the ARE transcription and upregulates the expression of NQO-1. The key step for Nrf2 translocation is its release from the Keap1 molecule, which binds and degrades Nrf2 in the cytoplasm. HSP20 might play some roles in this step. For example, previous study showed that transfection with a HSP20 phosphomimic into colonic smooth muscle cells can induce the phosphorylation of protein kinase C (PKC) [37], while PKC has been demonstrated to be necessary for the Nrf2 release from Keap1 in another study [38]. There are other possibilities for HSP20 acting on the release of Nrf2 with its inhibitor, such as through the cytoskeleton depolymerization. In early studies, researchers have proved that scaffolding of Keap1 to the actin cytoskeleton is key to the release of Nrf2 in cytoplasm [39], while phosphopeptide analogs of HSP20 can induce the depolymerization of cytoskeleton [40]. It is of worth to develop new investigations to testify these assumptions, to facilitate the understanding of the initial step of Nrf2 releasing from its complex inhibitor.

Though the phosphorylated HSP20 is the active form to exerts protective function, the unphosphorylated form might also possess some similar properties. In current study, cell apoptosis assay showed that even transfecting with the unphosphorylatable HSP20 still render the cells more resistant to the H_2_O_2_-induced cell necrosis as observed in the vector-transfected cells, which may imply that even the unphosphorylated HSP20 protein may also, to some degree, exert protective functions. Furthermore, in prior to H_2_O_2_ stimulation, transfection with the unphosphorylatable mutant HSP20 can increase the intracellular NQO-1 protein level but fail to elevate neither the NQO-1 mRNA nor the intranuclear Nrf2 level, which hint that there might be a favorable influence on the NQO-1 protein metabolism exerted by unphosphorylated HSP20 protein. These intriguing findings requires further substantiating in future studies to explore the biological function of unphosphorylated HSP20 with more precise and direct manipulation on the phosphorylation status of this small heat shock protein.

There is some limits in this study. Firstly, though BEAS-2B cell line is originated from human bronchial epithelial cells, the cells are immortalized and act and function in a different way that the primary human bronchial epithelial cells do in response to the environmental stress. Secondly, mere gain-of-function experiments and without loss-of-function experiments were not solid enough to demonstrate the contribution of HSP20 to the cell survival and oxidants metabolism. Finally, detecting the nuclear Nrf2 level but not its activity is somehow less convincing to demonstrate the participation of Nrf2-NQO-1 pathway in the HSP20 function. Therefore, it is necessary to confirm the protective role of HSP20 against oxidative stress and the mechanism(s) of action in primary human AECs and involve more comprehensive approaches in future studies.

## Conclusions

Our findings demonstrate that oxidative stress-induced expression of HSP20 and its phosphorylation in AECs may enhance the translocation of Nrf2 into nuclear and subsequently increase the expression of anti-oxidant enzyme, NQO-1. By tuning this oxidant-antioxidant balance, HSP20 exerts its protective functions on airway epithelial cells against oxidative damages. Consequently, HSP20 may serve as a new potential therapeutic target for chronic oxidative stress-related airway diseases, such as asthma and COPD*.*

## Materials and methods

### Chemicals and materials

Protease inhibitor cocktail tablets were from Roche. Anti-HSP20 antibody (ab184161) and anti-phospho-HSP20 antibody (ab58522) were from Abcam plc.® (Abcam China Co. Ltd., Shanghai, China). Anti-NQO-1 (62262), anti-Nrf2 (12721), anti-actin (4970), anti-GADPH (5174), and anti-lamina A/C (2032) antibodies were all from CST (Shanghai) Biological Reagents Co. Ltd. (Shanghai, China). The mouse Elisa kit for albumin (ab108792) was from Abcam plc.® (Abcam China Co. Ltd., Shanghai, China) and for E-cadherin (EMCDH1) was from Thermo Fisher Scientific China Co. Ltd. (Shanghai, China). All antibodies and reagents were validated by the corresponding vendors.

### Animals

Six-week-old female Balb/c mice weighting 18–20 g were purchased from SLAC Laboratory Animal Co. Ltd. (Shanghai, China) and bred under specific-pathogen-free conditions. The protocol was approved by the Shanghai General Hospital Institutional Review Board (Permit Number: 2010KY047).

### In vivo* ozone exposure*

As previously described [41], ozone (O_3_) was generated by an electronic O_3_ generator (AB Aqua MedicGmbH, Bissendorf, Germany) and the O_3_ concentration in the exposure chamber was automatically maintained at 2.0 ppm by an O_3_ sensor (Ozone Switch™, KWJ Engineering Inc., Newark, NJ). In the preliminary experiment, equal number of mice (n = 3) were respectively exposed to filtered air, 0.5 or 2.0 ppm O_3_ for 1 h and the expression of HSP20 in lungs were measured by immunohistochemistry (IHC) staining. In subsequent experiments, mice first received O_3_ preconditioning (n = 10): repeated exposures to 0.5 ppm O_3_ for 1 h per day, on every other day, for seven days, while control mice (n = 10) received repeated filtered air exposures at matching timepoints. Forty-eight hours after the exposures, these preconditioned mice as well as their counterparts were further stimulated with a single exposure of 2.0 ppm O_3_ (3 h, n = 5) or filtered air (3 h, n = 5). Mice were moved to their cages with fresh air, food and water immediately after each O_3_ exposure. Biological evaluations were conducted 24 h later. A schematic diagram of animal experimental protocol can be found in Fig. 1.

### Bronchoalveolar lavage (BAL)

After overdose sacrifice (pentobarbital, 100 mg/kg), mice were intubated and BAL fluid samples were collected, followed by counting the total and the differential cells, as previously described [42]. Concentrations of albumin and E-cadherin in cell-free BAL fluid were measured with commercial ELISA kits (R&D Systems China Co. Ltd., Shanghai, China), following the manufacturer’s instructions.

### Lung histology

The left lung lobe was fixed in 10% neutral-buffered formalin solution and embedded with paraffin. Lung Sects. (5 μm) underwent hematoxylin and eosin (H&E) staining. The infiltration of inflammatory cells in peribronchial and perivascular area were evaluated respectively according to a 0 ~ 3 scoring system and the average of both scores was calculated, as previously described [41, 43].

The epithelial cell density was then calculated manually by counting the total epithelial cells per millimeter epithelium basal lamina in the proximal and distal airway segments, on the H&E stained sections, using Image J software.

### Immunohistochemical (IHC) staining

Lung sections were deparaffinized and rinsed with 3% hydrogen peroxide (H_2_O_2_) for 30 min, followed by incubating in 5% goat serum albumin for 30 min to block nonspecific protein binding. Then, the sections were incubated overnight at 4 °C with rabbit anti-HSP20 antibody (1:200, dilution; Abcam China Co. Ltd., Shanghai, China), followed by incubating biotin-labeled goat anti-rabbit IgG (1:500 dilution; Abcam China Co. Ltd., Shanghai, China) and horseradish peroxidase-conjugated streptavidin for 15 min, respectively. Eventually, the slides were incubated in DAB solution for 10 min at 37 °C, counterstained by hematoxylin, and mounted. The sections were observed and the photographs were acquired by a light microscope (Leica DM4000B, Germany) with 200 × magnification. Integrated optical density (IODs) of photographs were assayed by using Image J.

The percentage of HSP20-positive epithelial cells were calculated by dividing the number of HSP20-positive epithelial cells by the number of total epithelial cells on a certain length of a distant bronchus. The absolute number of HSP20-positive epithelial cells per millimeter epithelium basal lamina in the proximal and distal airway segments were also counted.

### Western blot

For in vivo study, the expression of phosphorylated (phospho-) and total HSP20 protein, and the actin (control) in lung tissues were measured. For in vitro study, the expression of NQO-1, phospho- and total- HSP20, and the GAPDH (control) in whole cell lysis, Nrf2 and Lamina A/C (control) in nuclear lysis were similarly measured. Briefly, total protein was extracted from lung tissues, by using RIPA buffer (CST (Shanghai) Biological Reagents Co. Ltd., Shanghai, China), and from whole cells or nuclear pellets by using a nuclear extraction kit from Active Motif (distributed by Shanghai Universal Biotech Co. Ltd., Shanghai, China) as per manufacturer’s instructions and quantified. After centrifugation (15,000 × *g*; 10 min; 4 °C), the protein was subjected to the subsequent SDS–PAGE (Bio-Rad Laboratories Shanghai Co. Ltd., China). After electrophoresis and transfer, the membranes were blocked and incubated with primary antibodies (for detection of total-HSP20, the phospho- HSP20 blotted gels were stripped and then reprobed). The binding of the primary antibody was detected by infrared dye-conjugated secondary antibodies and Odyssey® system (Li-Cor Inc., NE), and bands densitometry were quantified.

### RT-qPCR

As previously described [42], total RNA was extracted in lung tissue or cultured cells and used to synthesize cDNA. Transcript levels were determined using SYBR Green PCR Master Mix Reagent (Qiagen, Stockach, Germany). The relative abundance of mRNA of HSP20 (human and mouse) and NQO-1 (human and mouse) were normalized to β-actin. The sequences of primers used in the PCR (synthesized by Invotrigen®, Thermo Fisher Scientific China Co. Ltd., Shanghai, China) are listed in Table 1.

**Table 1:** Sequences of primers used in RT-qPCR

Primer	Forward primer	Reverse primer
HSP20 (Mouse)	5′-TCTTTGACCAGCGTTTCGGC-3′	5′-GTATCGGCGGTGGAACTCTC-3′
NQO-1 (Mouse)	5′-AGCCAATCAGCGTTCGGTAT-3′	5′-GCCTCCTTCATGGCGTAGTT-3′
HSP20 (Human)	5′-CCAGATATCCTCGGCAACCC-3′	5′-TGGGAGAGTAAGCCTGGGAA-3′
NQO-1 (Human)	5′-GCTGGTTTGAGCGAGTGTTC-3′	5′-CTGCCTTCTTACTCCGGAAGG-3′

### Measurements on oxidants

The content of Malondialdehyde (MDA) and the activity of glutathione peroxidase (GSH-Px) activity in lung tissue homogenates were measured with respective substrates (Nanjing Jiancheng Bioengineering Institute, Jiangsu, China) using a spectrophotometry-based method, as previously described [42].

### Cell culture

The human bronchial epithelial cell lines BEAS-2B and 16HBE were obtained from the American Type Culture Collection (distributed by Beijing Zhongyuan Ltd., Beijing, China). After validation for respective markers, BEAS-2B and 16HBE cells were incubated in RPMI 1640 medium containing 10% fetal calf serum (FCS) (Invitrogen®, Thermo Fisher Scientific China Co. Ltd., Shanghai, China) and antibiotics in a humidified atmosphere at 37 °C with 5% CO_2_. To achieve an air–liquid-interface (ALI), 16HBE cells were seeded into 24-transwell plates, and subjected to ozone exposure and H_2_O_2_ stimulation.

In ALI cultures, 16HBE cells were tentatively treated with varying concentrations of H_2_O_2_ (0, 25, 50, 100, 200, 400, and 800 μM) or exposed to 2.0 ppm ozone (identical with the in vivo stimulation) or filtered air for 0.5, 1, 2, and 4 h, followed by cell viability measurements. To evaluate oxidative stress, 16HBE cells were treated with H_2_O_2_ (50, 100, 200 μM) or 2.0 ppm ozone for 0.5, 1, 2 h, in prior to measurements for 4-Hydroxynonenal (4-HNE), a lipid peroxidation product(44), and 8-hydroxy-deoxyguanosine (8‑OHdG), a biomarker of oxidative DNA damage(45).

To explore the potential inducing effects of H_2_O_2_ on the expression of HSP20, BEAS-2B cells were seeded in 24-well (1 × 10^5^/ml, 200 μl) plates and treated with varying concentrations of H_2_O_2_ (0.1, 1.0, 10, 100 μM) for 1 h (h), and after a subsequent fresh culture for 0, 12 and 24 h, cell viability and HSP20 expressions (Western blot, immunocytochemical staining and RT-qPCR) were measured. Through this pilot experiment, 10 μM H_2_O_2_ followed by 12 h fresh culture was designated the condition for subsequent experiments.

### In vitro* ozone exposures*

Ozone generation and concentration monitoring instruments were identical with those utilized in in vivo experiment. Briefly, ALI-cultured 16 HBE cells in 24-transwell plates (2 × 10^5^/ml, 100 μl) were placed into a sterile chamber, where the input air or ozone was filtered before entering, for 0.5, 1, 2, and 4 h (h), after the medium were removed from the basal compartments. Ozone monitor probe was placed in a position level with the apical compartments. the chamber.

### Cell viability and apoptosis measurements

Cell viability was evaluated by using MTS for 16HBE culture and MTT assay for BEAS-2B culture. The apoptosis assay was performed on BEAS-2B cells stained with Annexin-V and PI as per manufacturer’s instruction (FITC Annexin V apoptosis detection kit I, BD pharmingen™, CA) utilizing flow cytometry (FACSCalibur™, CA) and CellQuest 3.0 software (Becton–Dickinson Biosciences Inc., San Jose, CA).

### DNA overexpression transfection

pEX-3-*HSP20* and pEX-3-*HSP20*(Ala) plasmids, encoding the wild-type HSP20 and the nonphosphorylatable form of HSP20 (Ala) (HSP20 clone generated from human HSPB genes, and constructed by Shanghai GenePharma Co. Ltd, Shanghai, China), were transfected into BEAS-2B cells using Lipofectamine® 2000 (Invitrogen®, Thermo Fisher Scientific China Co. Ltd., Shanghai, China) reagent (DNA: Lipofectamine 2000 = 1 μg:2.5 μL), along with the vector plasmid (pEX-3 only), respectively (see Additional file 1: Figure S1). The transfection efficiency using each expression plasmids is similar. The protein and mRNA expressions of HSP20 were measured in differently transfected cells (data not shown). Forty-eight hours after the transfection, cells were undergoing subsequent H_2_O_2_ stimulation and biological measurements thereafter.

### Intracellular ROS measurements

The intracellular ROS level was measured with a DCFH-DA (2′,7′-Dichlorodihydrofluorescein-diacetate) Reactive Oxygen Species Assay Kit (Beyotime, Nang Jing, China). Briefly, BEAS-2B cells were incubated with DCFH-DA (10 μM) at 37 °C with 5% CO_2_ for 30 min. Fluorescent intensity was then measured by using the Tecan Infinite® 200 microplate reader (LabX™, ON, Canada) with excitation/emission wavelength of 488/525 nm. All readings were taken in triplicates.

### Measurements on 4-HNE and 8‑OHdG

4-Hydroxynonenal (4-HNE) (Abcam, Cambridge, MA, USA) was measured in the supernatants of 16HBE culture by ELISA as per the manufacturer’s instructions. Intracellular levels of 8-hydroxy-deoxyguanosine (8-OHdG) was measured on the extracted DNA of differently stimulated 16HBE cells using the ELISA kits (Abcam, Tokyo, Japan) according to the manufacturer’s instructions. Briefly, DNA was extracted and purified with a TIANamp Genomic DNA Kit (TIANGEN BIOTECH (BEIJING) CO. LTD., Beijing, China) and then digested with DNase I (DN25-1G, Sigma-Aldrich) and Nuclease P1 (N8630, Sigma-Aldrich) before incubation with alkaline phosphatase (Sangon, Shanghai, China) at 1 unit AP/100 µg DNA at 37 °C for 30 min, and boiled for 10 min before 8-OHdG measurement. All experiments were performed three times independently.

### Statistical analysis

All the data were analyzed using statistical software SPSS 18.0 and expressed as means ± SD. In vitro experiments were repeated for three times. Differences among groups were determined using one-way ANOVA, followed by Tukey as a post hoc test. Comparisons between two groups were performed using an unpaired Student’s *t*-test. A *p* < 0.05 was considered to be statistically significant.

## Supplementary information

**Additional file 1: Figure S1.** The structure HSP20-overexpression plasmids and the confirmation of their transfection. A: Diagram of recombinant pEX-3 plasmids. Human HSP20 cDNA and its mutants of TCA-GCA (replaced Ser16 with Ala to block phosphorylation) were inserted, respectively, into a pEX-3 plasmid, namely pEX-3-HPS20, pEX-3-HSP20(Ala), respectively. The mutated nucleotides were identified in bold. B: The HSP20 expressions in differently transfected cells were confirmed at both mRNA (B) and protein level (C).

## Data Availability

The datasets used and/or analysed during the current study are available from the corresponding author on reasonable request.

## Abbreviations

AECs: Airway epithelial cells
ASM: Airway smooth muscle
ARE: Antioxidant response element
BALF: Bronchoalveolar lavage fluid
COPD: Chronic obstructive pulmonary disease
GSH-Px: Glutathione peroxidase
GST: Glutathione S-transferases
H&E: Hematoxylin and eosin
hrs: Hours
H_2_O_2_: Hydrogen peroxide
ICC: Immunocytochemistry
IHC: Immunohistochemistry
MDA: Malondialdehyde
MTT: Methyl thiazolyl tetrazolium
NQO-1: NAD(P)H quinone oxidoreductase 1
Nrf2: NF-E2 related factor 2
O_3_: Ozone
ROS: Reactive oxygen species
