# Correction to: Inducible expression of heat shock protein 20 protects airway epithelial cells against oxidative injury involving the Nrf2-NQO-1 pathway

**DOI:** 10.1186/s13578-020-00510-3

**Published:** 2020-12-19

**Authors:** Aihua Bao, Aying Ma, Hui Zhang, Lihua Qiao, Suqin Ben, Xin Zhou, Min Zhang

**Affiliations:** 1 Department of Respiratory and Critical Care Medicine, Shanghai General Hospital, Shanghai Jiao Tong University School of Medicine, 100 Haining Road, Shanghai, 200080 China; 2 Department of Respiratory and Critical Care Medicine, The First Affiliated Hospital of Zhengzhou University, Henan, China; 3 Department of Gynecology, The Fourth People’s Hospital of Shanghai, Tong Ji University, Shanghai, China

## Correction to: Cell Biosci (2020) 10:120 http://doi.org/10.1186/s13578-020-00483-3

Following publication of the original article [1], one of the grant number was missed to include in the Funding section.

The updated Funding is given below.
