# Supplementary material for: Inducible expression of heat shock protein 20 protects airway epithelial cells against oxidative injury involving the Nrf2-NQO-1 pathway
Source: Cell Biosci. 2020 Oct 19;10:120. doi: 10.1186/s13578-020-00483-3 (PMC7574176; doi:10.1186/s13578-020-00483-3)
Supplement: Supplementary file 1 — Additional file 1: Figure S1. The structure HSP20-overexpression plasmids and the confirmation of their transfection. A: Diagram of recombinant pEX-3 plasmids. Human HSP20 cDNA and its mutants of TCA-GCA (replaced Ser16 with Ala to block phosphorylation) were inserted, respectively, into a pEX-3 plasmid, namely pEX-3-HPS20, pEX-3-HSP20(Ala), respectively. The mutated nucleotides were identified in bold. B: The HSP20 expressions in differently transfected cells were confirmed at both mRNA (B) and protein level (C). [file 13578_2020_483_MOESM1_ESM.pdf]

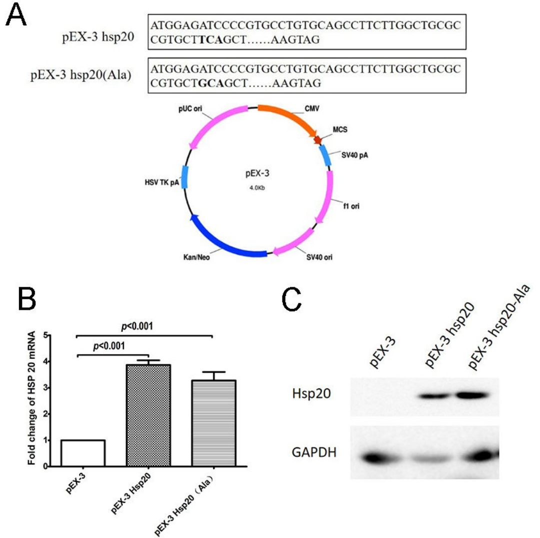
A
pEX-3 hsp20
ATGGAGATCCCCGTGCCTGTGCAGCCTTCTTGGCTGCGC
CGTGCTTCAGCT……AAGTAG
pEX-3 hsp20(Ala)
ATGGAGATCCCCGTGCCTGTGCAGCCTTCTTGGCTGCGC
CGTGCTGCAGCT……AAGTAG
pUC ori
CMV
MCS
SV40 pA
pEX-3
4.0Kb
HSV TK pA
f1 ori
Kan/Neo
SV40 ori
B
p<0.001
p<0.001
Fold change of HSP 20 mRNA
5
4
3
2
1
0
pEX-3
pEX-3 Hsp20
pEX-3 Hsp20（Ala）
C
pEX-3
pEX-3 hsp20
pEX-3 hsp20-Ala
Hsp20
GAPDH
